# Specific mechanism of *Acidithiobacillus caldus* extracellular polymeric substances in the bioleaching of copper-bearing sulfide ore

**DOI:** 10.1371/journal.pone.0213945

**Published:** 2019-04-12

**Authors:** Shoushuai Feng, Kaijun Li, Zhuangzhuang Huang, Yanjun Tong, Hailin Yang

**Affiliations:** 1 School of Biotechnology, Jiangnan University, Wuxi, People’s Republic of China; 2 The Key Laboratory of Industrial Biotechnology, Ministry of Education; Wuxi, People’s Republic of China; 3 Key Laboratory of Carbohydrate Chemistry and Biotechnology (Jiangnan University) Ministry of Education; Wuxi, People’s Republic of China; 4 State Key Laboratory of Food Science and Technology, Jiangnan University, Wuxi, People’s Republic of China; 5 School of Food Science and Technology, Jiangnan University, Wuxi, People’s Republic of China; Institute of Materials Science, GERMANY

## Abstract

This study aimed to reveal the specific mechanism of extracellular polymeric substances (EPS) in the bioleaching of copper-bearing sulfide ore by moderately thermophilic bacterium *Acidithiobacillus caldus*. The bioleaching performance of blank control (BC), planktonic cell deficient (PD), attached cell deficient (AD), and EPS deficient (ED) systems were compared, to investigate the specific functions of “non-contact” and “contact” (including direct contact and, EPS-mediated contact) mechanisms. The detailed mechanics of bioleaching were studied using *μ*_*x*_ of cell growth, scanning electron microscopy (SEM), X-ray diffraction (XRD), and Fourier transform infrared spectroscopy (FTIR). The *μ*_*x*_ of cell growth demonstrated that EPS favors planktonic and attached cell growth. SEM observation revealed that intensive micro-pores on slag benefitted from the “EPS-mediated contact” mechanism. XRD identification indicated that additional chemical derivatives were produced via “EPS-mediated contact” mechanism, because of the active iron/sulfur metabolism. FTIR analysis revealed that the absorption peaks of C-O-S, sulfate, and S = O, which are closely associated with sulfur metabolism, have significant influences of EPS secretion. Taken together, the “EPS-mediated contact” mechanism contributed to almost half of the “contact” mechanism efficiency and a quarter of the total bioleaching efficiency. The proposed specific mechanism of EPS can deepen our understanding of similar bioleaching processes.

## Introduction

With continuous decline in copper rich ore reserves, reprocessing of low-grade copper-bearing sulfide ores, accounting for >70% of the global copper reserves, has become inevitable [1–3]. However, metal extraction from these low-grade ores using traditional smelting techniques is uneconomical. Therefore, most low-grade ores have been discarded [4–6]. Recently, owing to advantages such as simple operation, low infrastructure investment, and reduction in environmental pollution, bioleaching has become a dominant technology for recycling metal resources from discarded ores, compared with conventional pyrometallurgy [7–12]. However, as a primary sulfide ore, copper-bearing sulfide ore is difficult to dissolve through bioleaching process, due to its complicated composition, passivation effect, and high lattice energy (tetrahedral crystal structure) [2, 5]. Thus, improving the efficiency of copper-bearing sulfide ore bioleaching processes has attracted increasing attention in recent years.

To improve the rate of copper dissolution during bioleaching, it is necessary to gain a greater understanding of bioleaching mechanisms. Several investigations of the processes involved in “contact” and “non-contact” bioleaching mechanisms have proposed models to explain various aspects of the bioleaching process, including adsorption behavior and dissolution kinetics during sulfide ore bioleaching [13–16]. The “contact” mechanism, derived from the adsorption behavior of attached cells and leachates, is a prerequisite for initiating adhesion and subsequent iron/sulfur metabolism [17–19]. The “noncontact” mechanism, on the other hand, operates via with the chemical action of acidic ferric sulfate solutions produced from the bacterial oxidation of ferrous iron, which is normally present in these environments [20]. Recent studies have recognized the important role of extracellular polymeric substances (EPS) in bioleaching, especially at the mineral solution interface. It is well known that bacterial EPS are secreted into the cell envelope. These EPS contain polysaccharides, proteins, and lipids, which are beneficial for biofilm formation [21, 22]. It has been previously suggested that EPS of the iron-oxidizer *Acidithiobacillus ferrooxidans* might combine with Fe^3+^ and form the complex EPS-Fe^3+^ compounds [23]. The synthesized and secreted EPS of the mesophilic *A*. *thiooxidans* favor a stronger hydrophobic interaction in cell adhesion during bio-oxidative/bioleaching processes [24]. EPS was recognized as the direct evidence of colonization and biofilm formation of the extremely acidophilic archaeon *Ferroplasma acidiphilum* BRGM4 biofilms during attachment to the cracks/defects of pyrite surfaces [25]. However, the “EPS-mediated” mechanism for coupling with the moderately thermophilic sulfur-oxidizer *A*. *caldus*, which is widely recognized as the dominant strain in most bioleaching processes, is sophisticated and has not been completely elucidated thus far.

In the authors’ previous study, *A*. *thiooxidans* ZJJN-3 secreted a thick capsule and EPS-like complex, which exhibited good potential for adsorption behavior [26]. The “contact” mechanism was strengthened by the adapted adsorption behavior in the bioleaching of copper-bearing sulfide ore by *A*. *thiooxidans* and *A*. *ferrooxidans* [27]. The effects of the “non-contact” mechanism on community structure during chalcopyrite bioleaching by *Acidithiobacillus* sp. have also been studied [28].

In this study, the typical moderately thermophilic strain-*A*. *caldus* was used to investigate the specific mechanisms underpinning the “EPS-mediated contact” in the bioleaching of copper-bearing sulfide ore. First, the effects of EPS on sulfur/iron metabolism and the *μ*_*x*_ of cell growth were compared. The effects of the “EPS-mediated contact” mechanism on ore characteristics, such as slag morphologies, composition, and their complex composition and functional group differences were then investigated using scanning electron microscopy (SEM), X-ray diffraction (XRD) and Fourier transform infrared spectroscopy (FTIR), respectively. Finally, the contribution of “EPS-mediated” processes to the mechanisms of bioleaching was determined.

## Materials and methods

### Strain and growth conditions

The typical moderately thermophilic sulfur-oxidizer *A*. *caldus* CCTCC M2018054 (16S rRNA gene GenBank number: Z29975) was isolated from the leachate from the bioleaching of copper-bearing sulfide, in Fujian, China. *A*. *caldus* was cultured in modified media from Deutsche Sammlung von Mikroorganismen und Zellkulturen GmbH. The basal salts of the modified media were listed as follows (in g/L): (NH_4_)_2_SO_4_: 3.0; KH_2_PO_4_: 3.0; K_2_HPO_4_ ·3H_2_0: 0.5; MgSO_4_·7H_2_O: 0.5; KCl: 0.1; Ca(NO_3_)_2_: 0.01. The energy substrate consisted of12.5 g/L S^0^. Trace elements were listed as follows (in mg/L): Na_2_SO_4_: 50.0; FeCl_3_·6H_2_O: 11.0; H_3_BO_3_: 2.0; MnSO_4_·H_2_O: 2.0; ZnSO_4_·7H_2_O: 0.9; Na_2_MoO_4_·2H_2_O: 0.8; CoCl_2_·6H_2_O: 0.6; CuSO_4_: 0.5; Na_2_SeO_4_: 0.1. The above chemicals were purchased from the Sinopharm Group (Shanghai, China). The initial pH of the media was adjusted to 2.0. The strains were adapted by 2.0% w/v low-grade chalcopyrite at 45°C and 170 rpm and were incubated into fresh media once a month.

### Ore sample composition and pretreatment

The low-grade copper-bearing sulfide ore was collected from Dongguashan copper mine, Tongling, Anhui, China. The detailed structure and composition of the ore sample were assayed by atomic absorption spectrometry, (Spectr AA-220, Varian, USA), and results are given in Table 1. The ore sample was crushed in a grinding mill (Raymond 3R1410, Jinghua, Weifang, China). The ground ore was sieved through a 300 mesh grid, with a < 48 μm controlling particle diameter. The sieved ore sample was gently washed in a solution of 2 M HCl, distilled water, and pure ethanol to remove surface dust. Finally, the ore sample was dried at room temperature and stored in a vacuum desiccator.

**Table 1:** The main characteristics of ore sample used in the study ^a^.

Parameter and unit	Value and description
Cu (%)	1.01 ± 0.02
S (%)	12.8 ± 0.21
Fe (%)	32.5 ± 0.50
Ca (%)	3.70 ± 0.23
Mg (%)	3.73 ± 0.21
Al (%)	1.40 ± 0.12
Zn (%)	0.054 ± 0.01
Mn (%)	0.047± 0.01
Ni (%)	0.028 ± 0.005
Pb (%)	0.026 ± 0.005
As (%)	0.0042 ± 0.001
Particle diameter (μm)	< 48^b^
Mineral type	Poor copper-bearing sulfide

^a^ The ore sample was collected from the Dongguashan copper mine, Tongling, Anhui, China; the values of Ag, Au, Co, Cd and Hg were all below detection limitation (< 0.0002).

^b^ The ore sample was ground and sieved through a 300-mesh grid, which controlled the particle diameter <48 μm.

### Experimental procedure

#### Procedure for PD, AD, and ED systems

**Fig 1:**
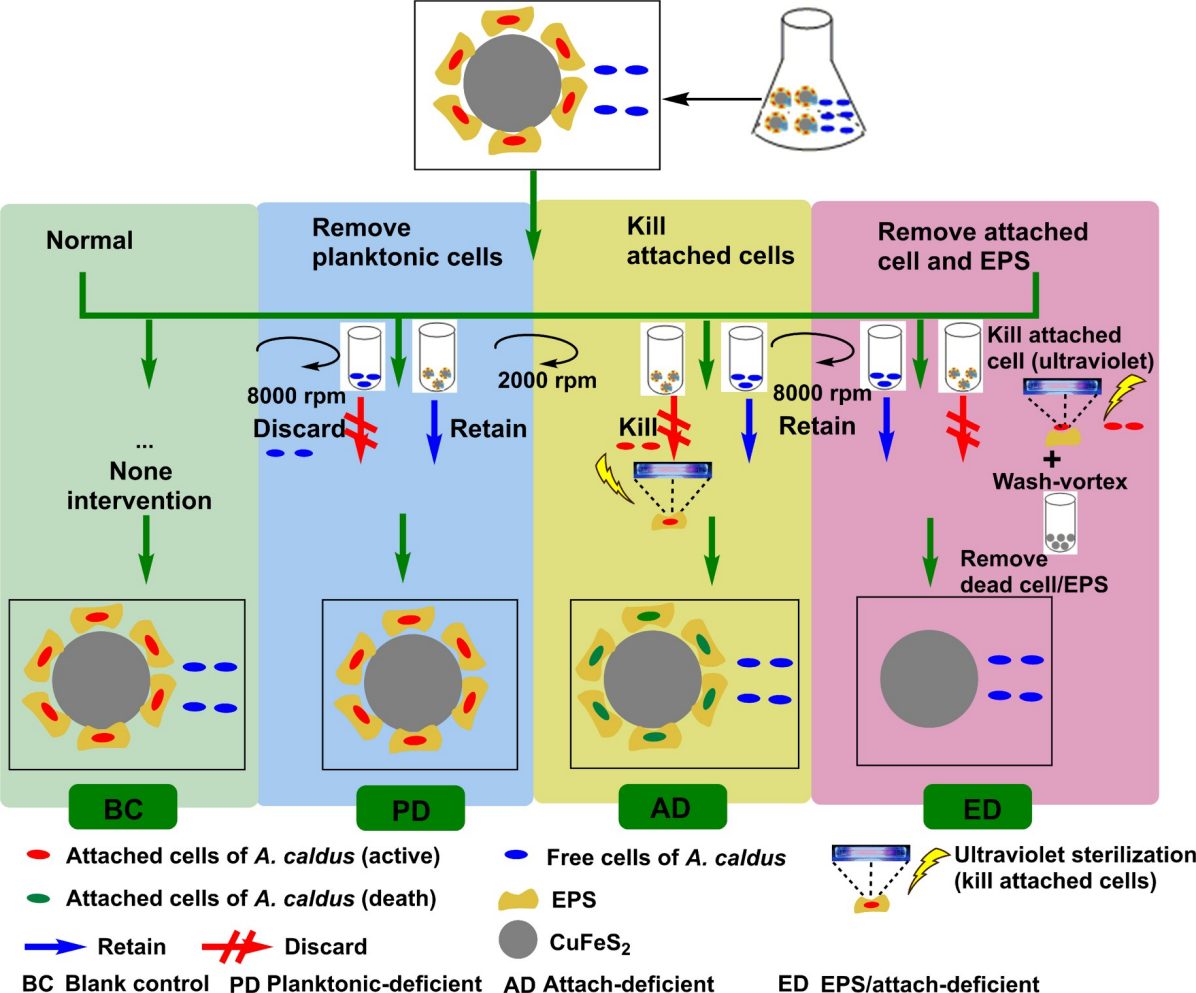
**The experimental setup of the different bioleaching procedures designed for this study:** *A*. *caldus* blank control system (BC); *A*. *caldus* planktonic cells-deficient system (PD); *A*. *caldus* attached cells-deficient system (AD); *A*. *caldus* EPS /attached cells-deficient system (ED).

Four related bioleaching experiments were designed, as follows: an *A*. *caldus* blank control system (BC); an *A*. *caldus* planktonic cells-deficient system (PD); an *A*. *caldus* attached cells-deficient system (AD); and an *A*. *caldus* EPS /attached cells-deficient system (ED). For the PD system, the leachate was allowed to rest without agitation for 1 h and the ore sample was then harvested by centrifugation at 380 × *g* for 2 min. The planktonic cells were then harvested by centrifugation at 6000 × *g* for 2 min and discarded. The ore samples were then re-combined with the original bioleaching supernatant free from planktonic cells (PD). Following the above procedures, no cells were counted by microscopy after one additional centrifugation (6000 × *g* for 2 min), indicating that all free cells in the supernatant had been removed. For the AD experiment, the ore sample was harvested by centrifugation at 380 × *g* for 2 min. The collected ore was then sterilized by killing attached cells with ultraviolet light for 1 h. The sterilized ore sample without attached cells was then transferred to its original system. For the ED experiment, the leached ore sample was sterilized with ultraviolet light to completely kill the attached cells, in a similar manner to the AD experiment. The ore sample was then suspended in 50 mL of fresh media and 1.0 g of 0.2 mm glass beads was added and agitated with a vortex (lab-dancer, IKA, Germany) for 5 min. The above centrifugation and agitation procedures were repeated. Almost no dissociative DNA remained in the supernatant after these procedures according to previous reports on this method, implying that all EPS and attached cells are removed [29]. The ore sample was then returned to its original bioleaching supernatant. The EPS and attached cells on the ore surface were thus removed completely. The experimental set-up for the four bioleaching systems is presented in Fig 1. The procedure outlined above was performed every two days.

#### Bioleaching experiment

The bioleaching experiments were carried out in 500 mL shaker flasks with 100 mL basal salts media. 2.0 g of low-grade chalcopyrite were added to each flask above the modified growth medium (with 12.5 g/L S^0^ as additional energy substrate). The initial biomass in each system was controlled at 5.0 × 10^7^ cells/mL after inoculation. The pH was maintained at 1.5 in order to maintain stable cell growth conditions, and was maintained at 1.5 through the use of 6 M HCl every two days. Considering that the excessive sulfate ions may participate in the formation of jarosite precipitate (which influences the later bioleaching and key parameter analysis), 6 M HCl was used to adjust the pH conditions. The bioleaching experiments were carried out at 45°C and 170 rpm. To compensate for daily evaporative loss from the leachate, 2.0 mL of sterile water were supplemented into each system once a day. The length of the whole bioleaching cycle was 30 days.

### Analytical methods

#### Measurement of key chemical parameters

The pH value was measured using a pH meter (FE28, Mettler, Switzerland). The Eh value was monitored by a Pt electrode (E-431Q, ASI, USA) with a calomel electrode (Hg/Hg_2_Cl_2_) as a reference. The concentration of sulfate ions was determined according to the chromic acid-barium colorimetric assay using a spectrophotometer (IV-1100D, Meipuda, China). The copper ion concentration was measured using a flame atomic absorption spectrophotometer (Spectr AA-220, Varian, USA). The ore sample was dried in a vacuum desiccator at 25°C and was then placed on insulated rubber tape for SEM (Quanta-200, FEI, Netherlands) observation of the mineralogical morphology of the residue surface, with a scanning rate set from 300 ns to 30 ns. The leached ore residue was gently washed with deionized water, dried at room temperature, and the dried ore residue was placed in the central hollow of the detection plate. The ore sample was scanned from 3 to 90° with a rate of 4°/min using an XRD (D8, AXS, Germany). The detailed data were then analyzed by the software MDI Jade 6.0 (Materials Data Ltd., USA) with the PDF card library. Approximately 2 mg of dried ore sample and 200 mg of KBr were ground in an agate mortar. A small sample was collected and formed into a thin cylinder shape by a squeezer. The cylinder was measured using an FTIR spectrometer (NEXUS, Thermo, USA), with the wavelengths 400–4000 cm^-1^ and an accuracy of 0.01 cm^-1^. The concentration of sulfate ions (in mg/L) was calculated as = 201.6 × OD_420 nm_− 26.029 (r^2^ = 0.998). The concentrations of ferrous and ferric ions were measured according to the *o*-phenanthroline spectrophotometry assay using a spectrophotometer. The concentration of ferrous ions (in mg/L) was calculated as = 5.077 × OD_508 nm_− 0.0765 (r^2^ = 0.999), the concentration of ferric ions (in mg/L) was calculated as = 5.102 × OD_508 nm_− 0.143 (r^2^ = 0.998) and the concentration of copper ions (in mg/L) was calculated as = 6.852 × λ_325 nm_− 0.0301 (r^2^ = 0.999).

#### Quantification of planktonic, attached, and total biomass

One milliliter of leachate from the bioleaching process was sampled and centrifuged at 380 × *g* for 2 min. The supernatant was collected, and the amount of planktonic biomass was measured using an optical microscope at 640 X magnification (Leica, Germany). Meanwhile, 5.0 mL of fresh basal media were added to re-suspend the bottom ore sample. 0.2 g of 0.2 mm glass beads were then added, and the mixture was agitated by a vortex for 5 min. The mixing and centrifugation processes were repeated once, to separate the attached cells from the ore surface. The cells in the supernatant were counted as attached biomass using a single span microscope and total biomass was calculated as the sum of the planktonic cells and the attached biomass.

## Results and discussion

### Effects of the “EPS-mediated contact” mechanism on sulfur/iron metabolism and cell growth (*μ*_*x*_)

#### Sulfur/iron metabolisms

**Fig 2:**
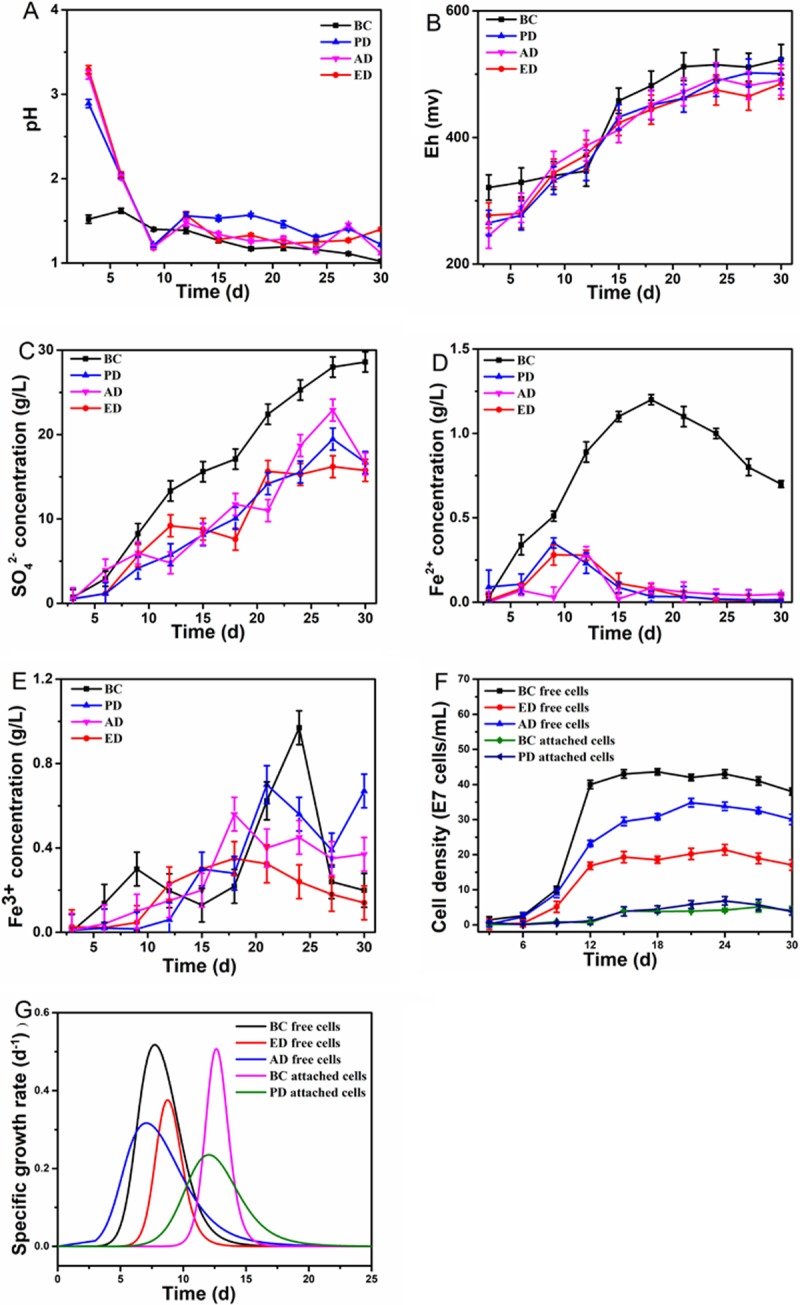
Changes in key chemical parameters in different deficient bioleaching systems. (A): pH; (B): Eh; (C): Sulfate ions; (D): Ferrous ions; (E): Ferric ions; (F) Planktonic and attached biomass; (G) *μ*_*x*_ of planktonic and attached cells. Note: the pH of leachate during bioleaching was measured before the pH-1.5 adjustment by 6 M HCl every two days.

The changes in chemical parameters due to iron/sulfur metabolism, such as the changes in sulfate, ferrous, and ferric ions, are shown in Fig 2. The main biochemical reactions in the bioleaching of copper-bearing sulfide ore are summarized in Equations (Eq) (1)–(6). Following the adaptive phase, the accumulated granular sulfur on the ore surface began to be oxidized by growing attached cells, as shown in Eqs (4) and (5). The pH therefore decreased and the sulfate ion content gradually increased. In the BC system, sulfur metabolism was active with a better comprehensive bioleaching mechanism, and the value of pH was maintained at about 1.50 in the early phase. Meanwhile, the values in deficient systems greatly fluctuated owing to the release of alkaline substances from the ore and poor sulfur oxidation efficiency. The final values of pH in the different systems were 1.02 (BC), 1.22 (PD), 1.12 (AD) and 1.40 (ED). These results demonstrate the importance of EPS for sulfur oxidation. The highest value of sulfate ion concentration was determined in the ED system (16.40 g/L), compared to 28.60 g/L (BC), 19.46 g/L (PD) and 18.70 g/L (AD) in the other systems. Exopolysaccharides have been recognized as essential to the granular sulfur oxidation process by *A*. *thiooxidans* [30]. These EPS transitioned from passive or inactive phases (containing CuS and S_n_^2−^) to active phases (containing increasing amounts of S^0^) on chalcopyrite surfaces. In the PD system, due to the lack of planktonic cells, the soluble intermediate sulfur (S_x_O^n^_y_) could not be oxidized, which resulted in inactive sulfur metabolism. In the AD system, the granular sulfur on the ore surface could not be used by attached cells and therefore formed the S^0^ passivation layer, inhibiting subsequent sulfur oxidization. Additionally, the final values of Eh in deficient systems (485–501 mV) with an inactive iron/sulfur ion level were slightly lower than those in the BC system (523 mV). The bioleaching efficiency of chalcopyrite by thermophiles (*Acidianus brierleyi*, *Sulfolobus metallicus* and *Metallosphaera sedula*) is closely associated with the redox status fluctuation [31].

The sulfur metabolism and iron metabolism interacted with each other, as shown in Eqs (1)–(4). The highest concentrations of ferrous ions in each system (in g/L) were 1.20 (BC), 0.35 (PD), 0.30 (AD) and 0.27 (ED). It has been reported that the adsorption behavior of attached cells in the early stage is beneficial to further concentrate ferric ions and attack sulfide ore, as shown in Eq (1) [20]. The concentration of ferrous ions showed the greatest reduction, reaching 77.5%, in the attached cells /EPS-deficient system. Similarly, the sulfur metabolism of another mesophilic sulfur-oxidizer, *A*. *thiooxidans*, was more significantly influenced by attached cells, compared to the iron/sulfur-oxidizer, *A*. *ferrooxidans* [27]. Ferric ions showed a similar trend to that of ferrous ions. The highest concentrations of ferric ions (in g/L) in each system reached 0.97 (BC), 0.70 (PD), 0.56 (AD) and 0.35 (ED). It has been noted that the additional ferric ions accelerate the EPS adhesion process owing to the stronger electrostatic interaction by the mesophilic *A*. *ferrooxidans* [32]. These results indicate that the “EPS-regulated mechanism” of adsorption behavior was favorable for enhancing iron metabolism. A detailed comparison of key biochemical parameters between the different systems is presented in Table 2.

**Table 2:** Comparison of key chemical and biological parameters between pre-leaching and after-leaching in BC, PD, ED and AD systems.

Parameter and unit		Pre- leaching	After-leaching			
			BC	PD	AD	ED
Chemical indexes	Sulfate ion (g/L)	0.42 ± 0.05	28.6 ± 1.2	16.7 ± 1.1	16.5 ± 0.8	15.7 ± 1.3
	Conversion ratio of sulfate ion %^a^	None	63.3 ± 2.7	37.0 ± 2.4	36.5 ± 1.8	34.7 ± 2.9
	Ferrous ion (mg/L)	None	162.5 ± 12.5	312.5 ± 21.2	356.3 ± 26.2	65.4 ± 4.5
	Conversion ratio of ferrous ion %^a^	None	2.17 ± 0.17	4.17 ± 0.28	4.75 ± 0.35	0.87 ± 0.06
	Ferric ion (mg/L)	None	199.8 ± 12.2	670.2 ± 23.5	370.3 ± 21.2	141.2 ± 12.6
	Conversion ratio of ferric ion %^a^	None	2.66 ± 0.16	8.94 ± 0.31	4.94 ± 0.28	1.88 ± 0.16
	Total iron (mg/L)	None	362.3 ± 21.2	982.7 ± 32.3	726.6 ± 34.2	206.6 ± 17.2
	Conversion ratio of total iron %^a^	None	4.83 ± 0.28	13.11 ± 0.43	9.69 ± 0.46	2.75 ± 0.23
	Total Copper (mg/L)	None	60.72 ± 3.0	40.20 ± 1.6	42.72 ± 1.7	28.20 ± 0.8
	Daily productivity	None	1.52 ± 0.08	1.01 ± 0.04	1.67 ± 0.07	0.71 ± 0.02
	Mineral color	Black	Tawny	Gray	Gray	Gray
Biological indexes	Planktonic biomass (10^7^ cells/mL)	5.0	38.1 ± 1.0	None	30.0 ± 1.2	17.1 ± 0.8
	*μ*_*max*_	None	0.52	None	0.37	0.32
	*μ*_*max*_ date	None	7.67	None	7.14	8.91
	Attached biomass (10^7^ cells/mL)	None	4.09 ± 0.3	3.76 ± 0.3	None	None
	*μ*_*max*_	None	0.51	0.23	None	None
	*μ*_*max*_ date	None	12.10	11.87	None	None
	Attached ratio (%)	None	9.7 ± 0.9	100.0 ± 0.3	None	None
	Total biomass (10^7^ cells/mL)	5.0	42.19 ± 0.6	3.76 ± 0.3	30.0 ± 1.2	17.1 ± 0.8
	Daily productivity (10^7^ cells/mL)	None	1.05 ± 0.15	0.09 ± 0.01	0.75 ± 0.03	0.43 ± 0.02

^a^ It represents soluble ion (sulfate, ferrous, ferric, and total iron ion) in bioleaching system.

$$CuFeS_{2}+4Fe^{3+}=Cu^{2+}+2S^{0}+5Fe^{2+}$$ (1)

$$CuFeS_{2}+4H^{+}+O_{2}=Cu^{2+}+2S^{0}+Fe^{2+}+2H_{2}O$$ (2)

$$4Fe^{2+}+4H^{+}+O_{2}=4Fe^{3+}+2H_{2}O$$ (3)

$$2S^{0}+3O_{2}+2H_{2}O=2SO_{4}^{2-}+4H^{+}$$ (4)

$$S_{x}O_{y}^{n}+O_{2}+H_{2}O\to SO_{4}^{2-}+H^{+}$$ (5)

$$3Fe^{3+}+2SO_{4}^{2-}+6H_{2}O+K^{+}=KFe_{3}{\left(SO_{4}\right)}_{2}{\left(OH\right)}_{6}+6H^{+}$$ (6)

#### Biomass and *μ*_*x*_ of cell growth

A deficiency in planktonic cells, attached cells or EPS, significantly influences the planktonic and attached biomass (Fig 2D). Compared to 4.36 × 10^8^ cells/mL of planktonic biomass measured in the BC system, the planktonic biomass in the AD and ED systems was greatly reduced to 3.48 × 10^8^ and 2.02 × 10^8^ cells/mL, respectively, indicating the strong microbial cooperation between the planktonic population and the attached population. The “contact” mechanism was inhibited and fewer energy substrates and nutrients, necessary for cell growth, were released. Sulfur granules were primarily oxidized to an intermediate form, such as ${\text{S}}_{4}{\text{O}}_{6}^{2-}{\text{or S}}_{4}{\text{O}}_{5}^{2-}$, with the assistance of adsorption behavior [2,17]. The reduced and soluble sulfur were then completely used up via a “non-contact” mechanism. In contrast, the attached biomass measured in the PD system was 0.58 × 10^8^ cells/mL, even higher than that of the BC system (0.51 × 10^8^ cells/mL). The reason for this is thought to be that attached cells can directly obtain energy substrate and nutrients from the ore. Moreover, without the competition from planktonic cells, all released energy substrate or nutrients from the “contact” mechanism were available for growth of the attached cells. These data are consistent with the changes in chemical parameters, which also indicated a stronger requirement for EPS and attached cells by *A*. *caldus*.

The *μ*_*x*_ of attached and planktonic cells were significantly influenced by the absence of planktonic cells, attached cells and EPS (Fig 2D). The *μ*_max_ of planktonic cells in the ED system was reduced from 0.52 d^-1^ to 0.37 d^-1^ (AD) and 0.32 d^-1^ (ED), while the *μmax* of attached cells was also reduced from 0.51 d^-1^ to 0.23 d^-1^ (PD). More attached cells adsorbed to the ore surface with a stronger adsorption performance due to the mediating role of EPS, resulting in abundant nutrients becoming available for cell growth. Moreover, timing of the peak *μ*_max_ was clearly delayed in the absence of EPS. The *μ*_max_ of planktonic cells was tested after 7.67 days in the BC system and 8.91 days in the ED system. The peak time for attached cells was 12.10 days in the BC system, which was similar to that for the PD system (11.87 d). The kinetics data were consistent with the above results for attached biomass, with all the results supporting the importance of the role of EPS in the improvement of planktonic and attached cell growth of *A*. *caldus*.

### Effects of the “EPS-mediated contact” mechanism on ore morphology

**Fig 3:**
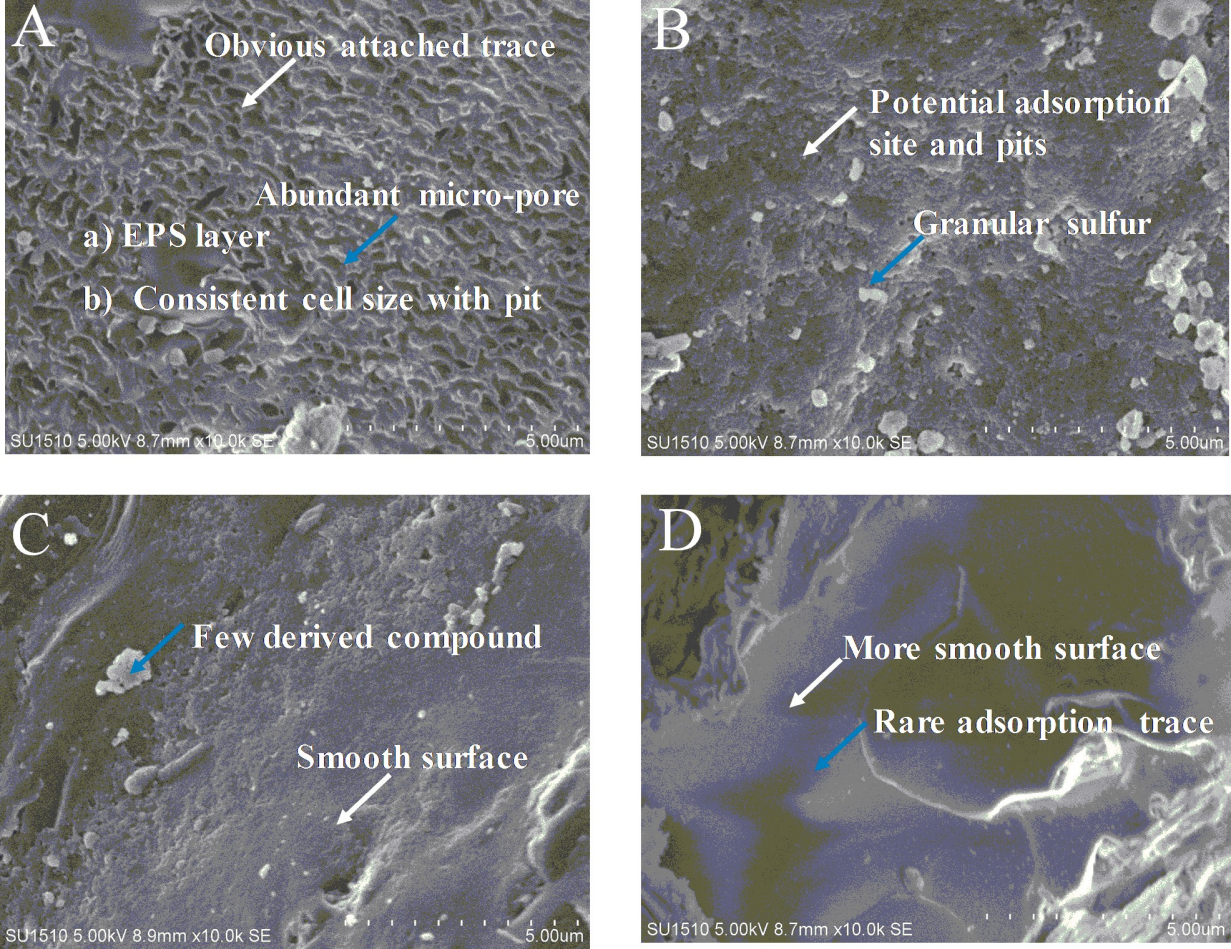
**Morphological surface differences of the ore samples between the different bioleaching systems:** (A): BC; (B): PD; (C): AD; (D) ED. The slag was dried at room temperature in a vacuum desiccator and observed via scanning electron microscopy at 10× k magnification (bar, 5 μm, 10× k).

To better understand the mineralogical effect of EPS, the morphologies of residues in the different bioleaching systems were observed by SEM (Fig 3). The morphological differences in the mineral residues between normal and deficient systems were found to be significant. In the BC system, intensive adsorption sites such as rills and micro-pores appeared on the ore surface, implying a stronger “contact” mechanism. Sulfur-oxidizers such as the mesophilic *A*. *thiooxidans* generally show a greater dependence on these for their adsorption behavior, as most of their main energy source (S^0^) is generated on the ore surface [28]. Interestingly, the size of the micro-pores was found to be similar to the cell size of *A*. *caldus* when observed using TEM (Transmitted Electron Microscopy) techniques (1.8–2.0 μm in length and 0.5–0.7 μm in width). In addition, TEM observation indicated that there was a clear EPS layer wrapped around the *A*. *caldus* cells, indicating a stronger effect of the “EPS-mediated contact” mechanism. It has been reported that the accumulation of slimy and soft EPS in *Sulfobacillus thermosulfidooxidans* biofilms stimulated adsorption behavior onto pyrite surfaces [33]. Moreover, due to the active chemical ion status, additional unknown derivatives were also produced and spread over the ore surface. In the PD system, due to the absence of planktonic cells, the cell growth of *A*. *caldus* only depended on the adsorption behavior of attached cells. The granular sulfur, which could not be completely utilized without attached cells, gradually accumulated on the ore surface. This conclusion was supported by the examination of the XRD patterns of the residues. In the AD system, the mineral surface was smoother and jarosite precipitation was minor. Owing to the absence of attached cells, the “contact” mechanism was greatly inhibited and there was insufficient energy substrate (ferrous ions) released from the ore for cell growth. Jarosite precipitation has been recognized as a major bottleneck in the later stage of the bioleaching of copper-bearing sulfides [23, 34]. The cleanest morphology was observed in the ED system, indicating from a different perspective, the importance of EPS for adsorption behavior.

### Effects of the “EPS-mediated contact” mechanism on ore components

**Fig 4:**
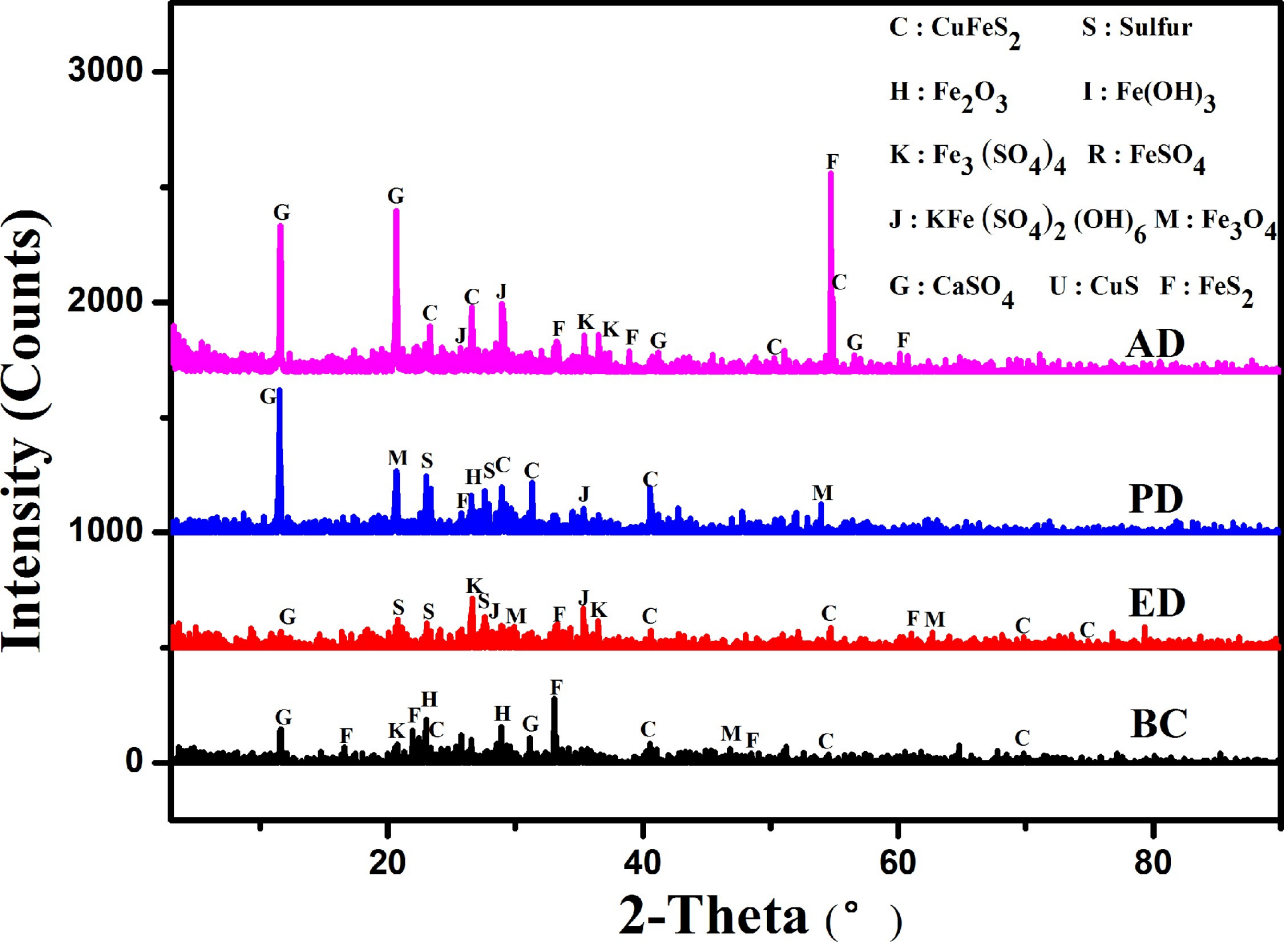
XRD analysis of the ore samples in different bioleaching systems. C:CuFeS_2_; S:Sulfur; H: Fe_2_O_3_; I: Fe(OH)_3_; K: Fe_3_(SO_4_) _4_; R: FeSO_44_; J: KFe_3_(SO_4_)_2_(OH)_6_; M: H: Fe_3_O_4_.

XRD analyses were performed to investigate the composition of ore samples in the different bioleaching systems (Fig 4). According to the XRD patterns, the main components were CuFeS_2_, KFe_3_(SO_4_)_2_(OH)_6_, S, Fe_7_S_8_, Fe_3_O_4_, FeS_2,_ and CaSO_4_·2H_2_O. In the BC system, the CuFeS_2_ peaks were wide, indicating the likely deformation of the crystalline structure during bioleaching. The assistance of the “EPS-mediated contact” mechanism accelerated iron/sulfur metabolism and produced more crystal forms [1,23]. In conjunction, large quantities of granular sulfur were not apparent in the XRD patterns, indicating complete utilization by attached cells. Higher temperatures increased the mass transfer rate during bioleaching by the moderately thermophilic *A*. *caldus*, which supports the improved efficiency in oxidizing S^0^ membranes [1,12]. In the PD system, the CuFeS_2_ peak remained high, which could be interpreted as incomplete dissolution of the ore during this bioprocess. In the AD system, the FeS_2_ peak was significant, indicating a less active iron/sulfur metabolism owing to the deficiency of attached cells. In contrast, there were relatively large amounts of precipitates such as jarosite and S^0^ in the residues of the deficient systems. It has been reported that, to some extent, elemental sulfur is generally coupled with amorphous iron or other oxy-hydroxides [23]. Meanwhile, the CaSO_4_·2H_2_O peak was significant in the PD and AD systems. Additionally, the presence of other unknown minor peaks in the analyses of these precipitates were speculated to be due to previous washes with deionized water prior to XRD detection. It can be interpreted that the XRD patterns of the solid residues indicate that the abundant inorganic ions and microbial organic complexes in the bioleaching systems contributed to more complicated derivatives [27]. The XRD analysis of the residues was consistent with the biochemical and photomicrograph analyses described above.

### Effects of the “EPS-mediated contact” mechanism on functional groups

**Fig 5:**
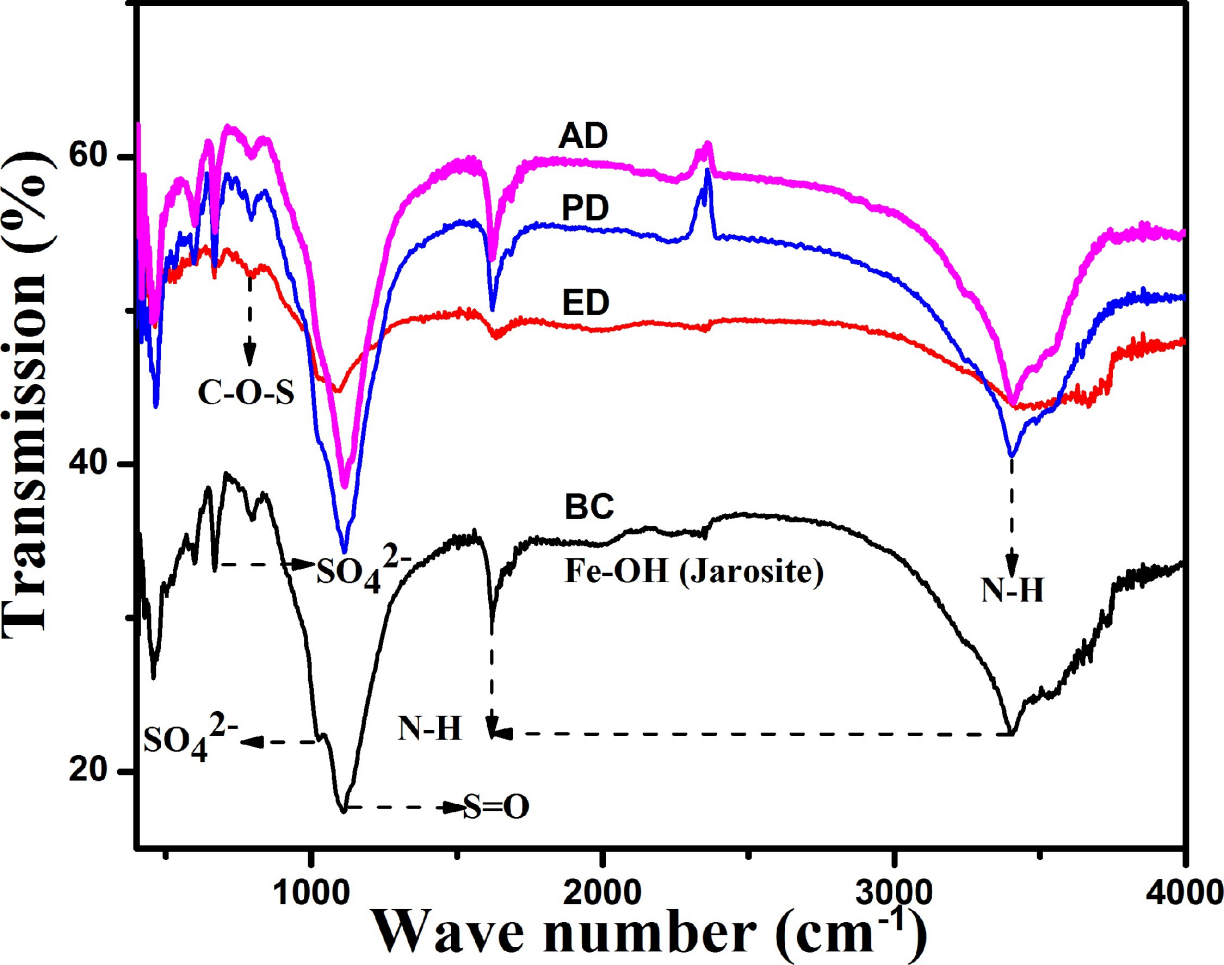
FTIR analysis of the ore samples in the different deficient bioleaching systems.

The functional groups of the ore in the infrared spectrum region were analyzed by FTIR analysis (Fig 5). The main absorption bands were 500, 680, 830, 1,000, 1,200, 1,600, 2,300, and 3,400 cm^-1^. In the BC system, the absorption band was complex owing to the presence of additional derivatives. It has been reported that the functional groups of *A*. *caldus*, such as S = O, -OH, -NH_2_, C = O, C-O, and -CONH_2_, are closely associated with the process of adsorption behavior [21]. The absorption peak at approximately 680 cm^−1^ was especially evident, while the single absorption peak around 1,000–1,100 cm^−1^ was split into two peaks. These absorption peaks are greatly influenced by variations in the performance of the sulfate ion or S = O. It has previously been reported that various intermediate metabolites, such as ${\text{S}}_{4}{\text{O}}_{6}^{2-}{\text{or S}}_{4}{\text{O}}_{2-}$ were produced during the oxidization process of the sulfur granules [6, 17]. In addition, a unique band was located at 830 cm^-1^, which was caused by C-O-S stretching vibrations. It has been reported that sulfate polysaccharide, another unique component of the bacterial biofilm EPS, is thought to be closely associated with the C-O-S stretching vibrations [33]. Additionally, the asymmetric stretching vibration of C-H and the band of the N-H group might also cause the peak shift around 1,600 and 3400 cm^-1^ [24]. In the ED system, the absorption peaks around 680 cm^-1^ and 1,000–1,100 cm^-1^ became smaller, owing to the inefficient sulfur oxidization caused by the absence of the “contact” mechanism. It has been suggested that several extracellular proteins with abundant thiol groups (Pr-SH) may be directly involved in sulfur activation in *A*. *ferrooxidans* by bonding to the elemental sulfur and forming Pr-Sn-SH complexes [32]. The missing absorption peak at 830 cm^-1^ in the ED system implies that the formation of sulfur-related derivatives such as sulfate polysaccharide (identified by the C-O-S stretching vibration) was greatly inhibited due to the EPS deficiency and inactive sulfur metabolism. In addition, the absorption peaks around 1600 cm^-1^ and 3400 cm^-1^, which are closely related to organic metabolism, were observed to have become less distinct owing to the lower organic content associated with poor cell growth.

#### Efficacy of the “EPS-mediated contact” mechanism for enhancing copper recovery

**Fig 6:**
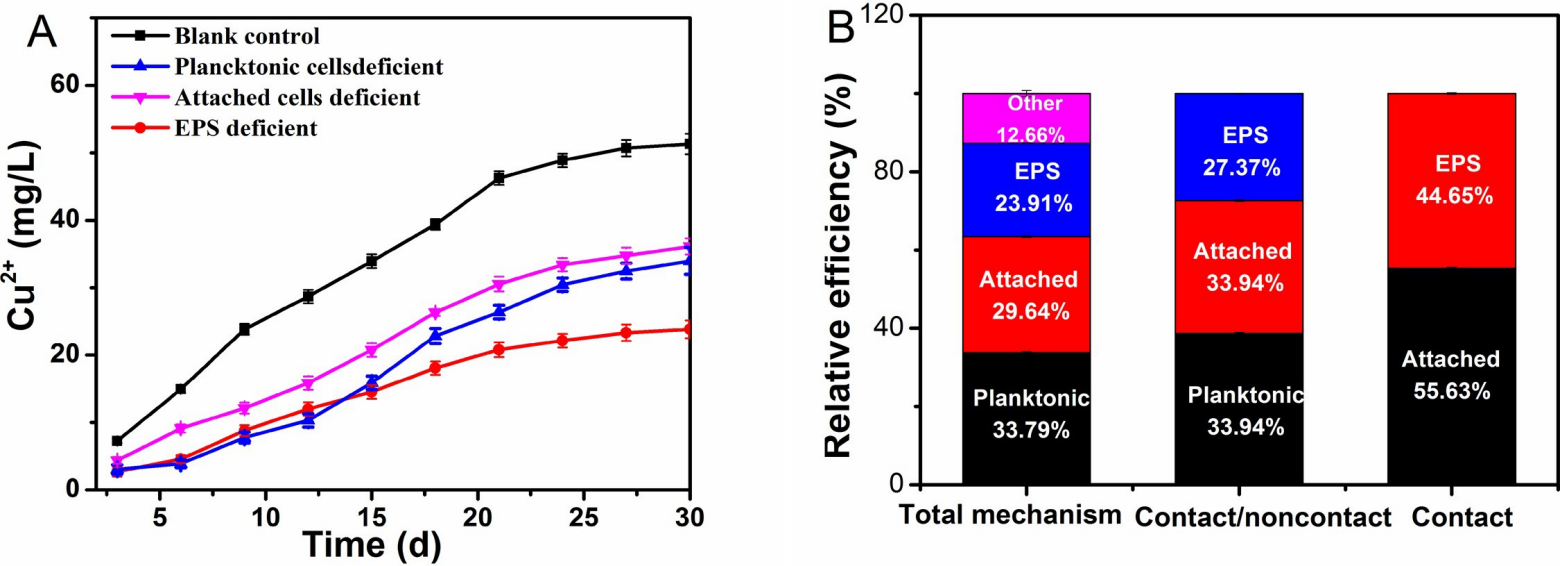
Changes in copper ions in different systems and relative bioleaching efficiencies of different mechanisms. (A): Copper ion; (B): Relative bioleaching efficiencies of different mechanisms.

The changes in copper ions in the different systems are shown in Fig 6A. Copper ions were gradually released into the leachate, concurrently with the oxidation of mineral iron and elemental sulfur during bioleaching. The final copper ion concentration in the deficient systems was greatly reduced because of the absence of the “non-contact” mechanism or “contact” mechanism (including the “direct contact” and “EPS-mediated contact” mechanism). The trend of the change was closely consistent with the biochemical parameters described above, which directly proves the function of EPS from the perspective of bioleaching efficiency. For the first time, the detailed contribution of the “EPS-mediated contact” mechanism to the bioleaching of copper-bearing sulfide ore using the moderately thermophilic *A*. *caldus* have been revealed, by systematically comparing the bioleaching performance differences between the BC, PD, AD and ED systems. We have shown that 23.91% of the bioleaching efficiency was contributed by EPS (“EPS-mediated contact” mechanism), while 29.64% and 33.79% of bioleaching efficiency were contributed by attached cells (through the “direct contact” mechanism) and planktonic cells (through the “non-contact” mechanism), respectively (Fig 6B). The “EPS-mediated contact” mechanism was responsible for 44.65% of the total efficiency of the “contact” mechanisms. Taken together, there was a close consistency of the copper ion extraction rates shown by the biochemical and mineralogical parameters, supporting the hypothesis that EPS played an important role in the bioleaching of copper-bearing sulfide ore by the moderately thermophilic *A*. *caldus*.

### Overall assessment of the “EPS-mediated contact” mechanism in copper-bearing sulfide bioleaching

**Fig 7:**
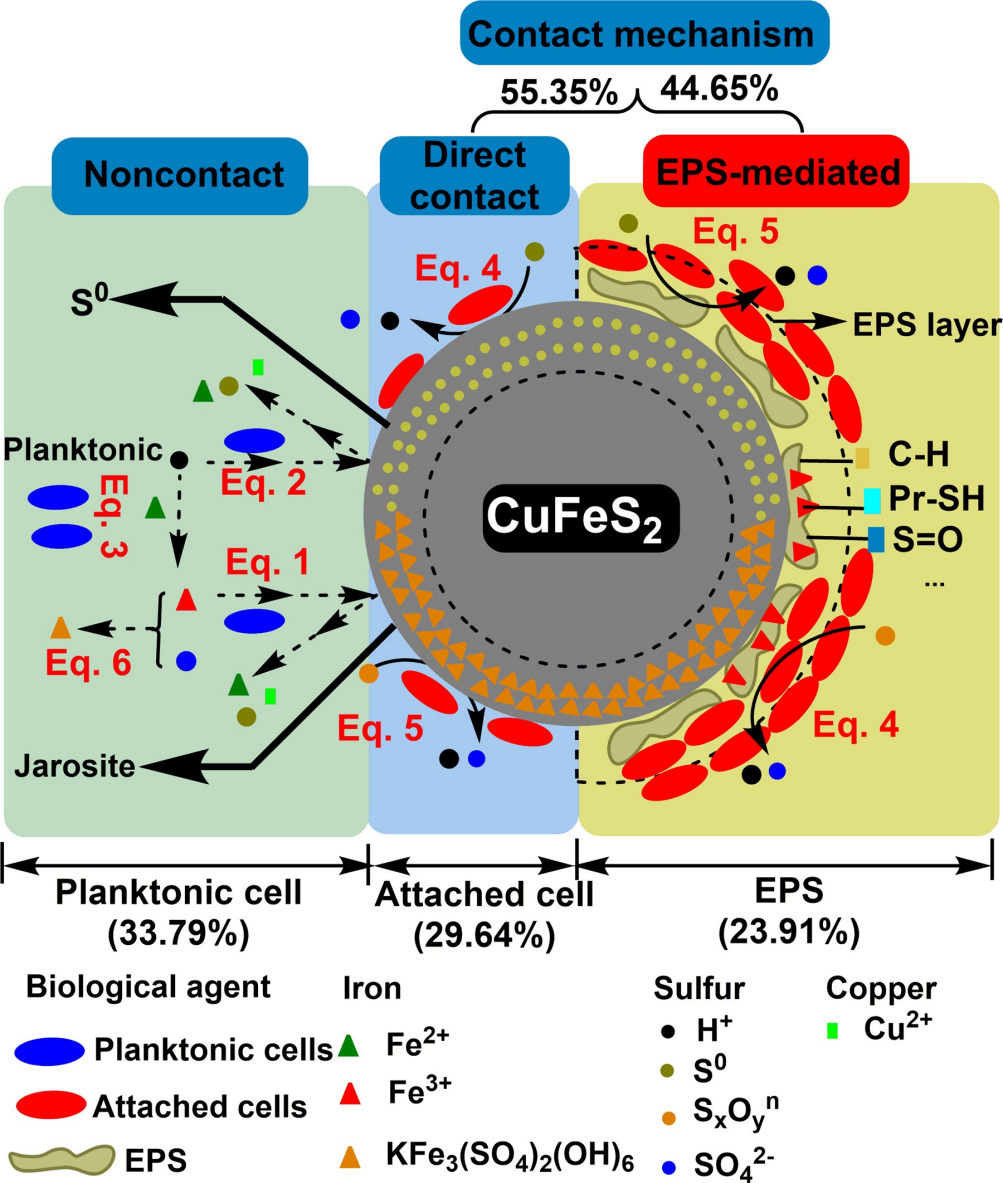
Overview of the specific function of the “EPS-mediated contact” mechanism in the bioleaching of copper-bearing sulfide ore by the moderately thermophilic *Acidithiobacillus caldus*.

The microenvironments involved in copper-bearing sulfide bioleaching may be divided into the solid-liquid interface and the liquid microenvironments, based on the biochemical reaction site. Attached cells and EPS complexes are apparently mainly active at the solid-liquid interface, while planktonic cells survive in the liquid microenvironment, which is greatly influenced by the former process. The “noncontact” “direct contact” and “EPS-mediated contact” mechanisms were derived from these three different microenvironments for the bioleaching of copper-bearing sulfide ore (Fig 7). The comparison of attached cells/EPS on the mineral surface between BC and ED system via confocal laser scanning microscope analysis (CLSM) was presented in Support Information.

The “noncontact” mechanism was performed by planktonic *A*. *caldus* cells. Planktonic cells oxidized ${\text{S}}_{4}{\text{O}}_{6}^{2-}{\text{or S}}_{4}{\text{O}}_{5}^{2-}$ in solution and released protons, as shown in Eq (5). The protons (H^+^) are essential for both the sulfur and iron metabolisms, as they attack the ore granules’ surface irregularities and release granular sulfur, as shown in Eq (2) [1, 6]. The protons also oxidize ferrous ions to ferric ions, as shown in Eq 3, and the ferric ions further attacked the ore, as shown in Eq (1). The dissolution of metal sulfides was therefore achieved through a combination of proton attack and ferric ion oxidation processes.

The “direct contact” mechanism was performed by the attached cells. During the sulfur oxidation process, sulfur colloids were oxidized to various intermediate compounds such as ${\text{S}}_{4}{\text{O}}_{6}^{2-}{\text{or S}}_{4}{\text{O}}_{5}^{2-}$ [9, 15]. In addition, redundant sulfur gathered as micro-particles (S_8_) and formed a barrier layer on the ore, blocking access to its surface. This reduced sulfur dissolved into the liquid microenvironment and was oxidized, as shown in Eq (5). The hydrogen ions then entered into the solid-liquid microenvironment and facilitated the “non-contact” mechanism. The reduced sulfur and hydrogen ions in the liquid microenvironment gradually initiated and enhanced the “non-contact t” mechanism [6, 35]. With the assistance of adsorption behavior, more hydrogen ions, ferrous ions, ferric ions, sulfur compounds, and planktonic biomass were generated in the bioleaching system. The biochemical activity of the bioleaching system was directly or indirectly affected by these oxidizing and reducing agents. Thus, to some extent, the adsorption behavior of attached cells acted as an initiator and accelerator of either the iron or sulfur metabolisms.

In the “EPS-mediated contact” mechanism coupling with EPS was first assessed from different mineralogical aspects. This mechanism contributed almost half of the “contact” mechanism efficiency and a quarter of the total bioleaching efficiency, indicating its vital role in bioleaching. EPS, which consist of polysaccharides, proteins and nucleic acids, generally serves as the reaction sites on the interface [14, 16]. EPS can help to form biofilms with attached cells, and can also concentrate ferric ions and protons. The EPS thickness and structure regulate the process of the “contact” mechanism. In general, the copper dissolution process takes place at the interface between the EPS and the ore surface. The released ferrous ions dissolve and participate in the ion cycle again, and the jarosite or iron precipitates combine with the EPS layer and act as weak diffusion barriers, interfering with the ion diffusion performance of the EPS layer [34]. In our previous study, with another two species of *Acidithiobacillus*, *A*. *ferrooxidans* and *A*. *thiooxidans* d as the model, the rate of EPS-mediated biofilm establishment was continuously enhanced during a directly adapted evolution process, and *A*. *thiooxidans* showed a higher dependence on the attached biofilm (contained EPS) mediated “contact mechanism” than that of *A*. *ferrooxidans* [27]. It was demonstrated that the adhesion force decreased in case the extracellular polymeric substances (EPS) had been removed. *L*. *ferrooxidans* exhibits the strongest force of adhesion to chalcopyrite and the largest contact angle, compared to that of *A*. *ferrooxidans* and *A*. *thiooxidans* [36]. It was also indicated that EPS from another bioleaching microorganism-*Acidianus* sp. DSM 29099 contains mainly protein and carbohydrate associated with planktonic cell surfaces or associated with biofilms on mineral surfaces, seems to mediate cell-cell and cell pyrite interactions during bioleaching [37]. The comparison between our study and other previous studies is given in Table 3.

**Table 3:** Comparison between our study and other previous related literatures.

Mineral type	Dominant strains	Operating conditions	Research emphasis/Adhesion trait	Ref.
Pyrite	*A*. *ferrooxidans*	NR^a^	A multilayered biofilm around with EPS was pivotal in “contact” mechanisms.	[9]
Pyrite	*Sulfobacillus* *thermosulfidooxidans*	pH 2.5 100–200 μm, 2.0% PD ^b^, 45°C.	Slimy and soft EPS accumulated in the biofilms and on the surface of pyrite to induce adhesion process.	[33]
Chalcopyrite, sulfur and pyrite	*A*. *ferrooxidans*	pH 2.3, 420–500 μm, 4.0% PD, 30°C.	Adsorption order: mineral-grown cell >ferrous/thiosulfate ion-grown cell.	[38]
Concentrate chalcopyrite	*A*. *caldus*, *L*. *ferriphilum*,	pH 2.0, <75 μm, 6.0% PD, 45°C.	Crude EPS components were identified as sugars, lipids, and uronic acid complex ferric ion.	[39]
Chalcopyrite	*A*. *ferrooxidans*, *A*. *thiooxdians* and *L*. *ferrooxidans*	pH 2.0, <75 μm, 30°C, 170 rpm.	*L*. *ferrooxidans* >*A*. *ferrooxidans* or *A*. *thiooxdians*; adhesion force was greatly reduced by removing EPS.	[40]
Chalcopyrite, pyrite, and sphalerite	*A*. *ferrooxidans*, *A*. *thiooxdians* and *L*. *ferrooxidans*	50–100 μm, 20.0% PD, 28°C.	Mineral-selection with the same strains; EPS was a key mediator.	[36]
Waste chalcopyrite, low-grade ore, pyrite and quartz	*A*. *ferrooxidans* and *L*. *ferriphilum*	pH 2.0, <75 μm, 0.5–2.0% PD, 0 K medium, 30°C.	“Contact mechanism” diversity between different minerals such as pyrite > chalcopyrite.	[35]
Chalcopyrite	*A*. *ferrooxidans*	<75 μm, 7.5% PD, 30°C.	EPS with additional Fe^3+^ increased the electrostatic interaction and initiated “contact mechanism”	[34]
low-grade copper-bearing sulfide ore	*A*. *ferrooxidans* and *A*. *thiooxdians*	pH 2.2, <48 μm, 3.0% PD, 30°C, 40 d.	A directly adapted evolution increased the contribution of “contact mechanism” (22.8% of *A*. *ferrooxidans*; 28.9% of *A*. *thiooxidans*).	[27]
low-grade copper-bearing sulfide ore	*A*. *caldus*	pH 2.2, <48 μm, 2.0% PD, 45°C, 40 d.	Assessment of the specific function/role of “EPS-mediated contact” mechanism (23.9% of total efficiency).	This study

^a^ NR: not reported;

^b^ PD: pulp density.

## Conclusions

The specific mechanism of “EPS-mediated contact” in the bioleaching of copper-bearing sulfide ores by the moderately thermophilic *A*. *caldus* was systematically assessed. The “EPS-mediated contact” mechanism has been shown to play a vital role in bioleaching and was responsible for almost half of the “contact” mechanism efficiency and a quarter of the total bioleaching efficiency. SEM observation revealed micropores of a size similar to the cell size on the ore surface, which is considered to have encouraged a stronger “EPS-mediated contact” mechanism. XRD analysis implied that additional chemical derivatives were produced with the assistance of the “EPS-mediated contact” mechanism. FTIR analysis indicated that the absorption peaks of the sulfate, C-O-S, and S = O, which are closely associated with sulfur metabolism, were significantly influenced by the presence of EPS. Taken together, our results support the hypothesis that the “EPS-mediated contact” mechanism provides essential support for the bioleaching of copper-bearing sulfide ore by the moderately thermophilic *A*. *caldus*. The work reported here may provide the basis for an improved understanding of sulfide ore bioleaching and similar bioprocesses for future research.

## Supporting information

S1 Fig CLSM analysis of attached cells and EPS on the mineral surface under different systems. BC blank control system; ED EPS deficient system. The leached ore samples were collected at different bioleaching periods. 20 μM SYTO 9 (L13152, Invitrogen, USA) was mixed with the sample (*λ*_*ex*_: 485 nm and *λ*_*em*_: 498 nm) with 200 μg/mL Alexa Fluor 594 ConA (C11253, Invitrogen, USA) (*λ*_*ex*_: 590 nm and *λ*_*em*_: 617 nm). After incubation in the dark for 30 min, the ore sample was collected by centrifugation, washed with 1 mL of PBS solution for 30 min. The treated ore sample was then incubated with PBS solution for 30 min and observed via CLSM (TSC SP8, Leica, Germany).
(DOCX)
