# Supplementary material for: Specific mechanism of Acidithiobacillus caldus extracellular polymeric substances in the bioleaching of copper-bearing sulfide ore
Source: PLoS One. 2019 Apr 12;14(4):e0213945. doi: 10.1371/journal.pone.0213945 (PMC6461249; doi:10.1371/journal.pone.0213945)
Supplement: S1 Fig — BC blank control system; ED EPS deficient system. The leached ore samples were collected at different bioleaching periods. 20 μM SYTO 9 (L13152, Invitrogen, USA) was mixed with the sample (λex: 485 nm and λem: 498 nm) with 200 μg/mL Alexa Fluor 594 ConA (C11253, Invitrogen, USA) (λex: 590 nm and λem: 617 nm). After incubation in the dark for 30 min, the ore sample was collected by centrifugation, washed with 1 mL of PBS solution for 30 min. The treated ore sample was then incubated with PBS solution for 30 min and observed via CLSM (TSC SP8, Leica, Germany). (DOCX) [file pone.0213945.s001.docx]

**Support Information**


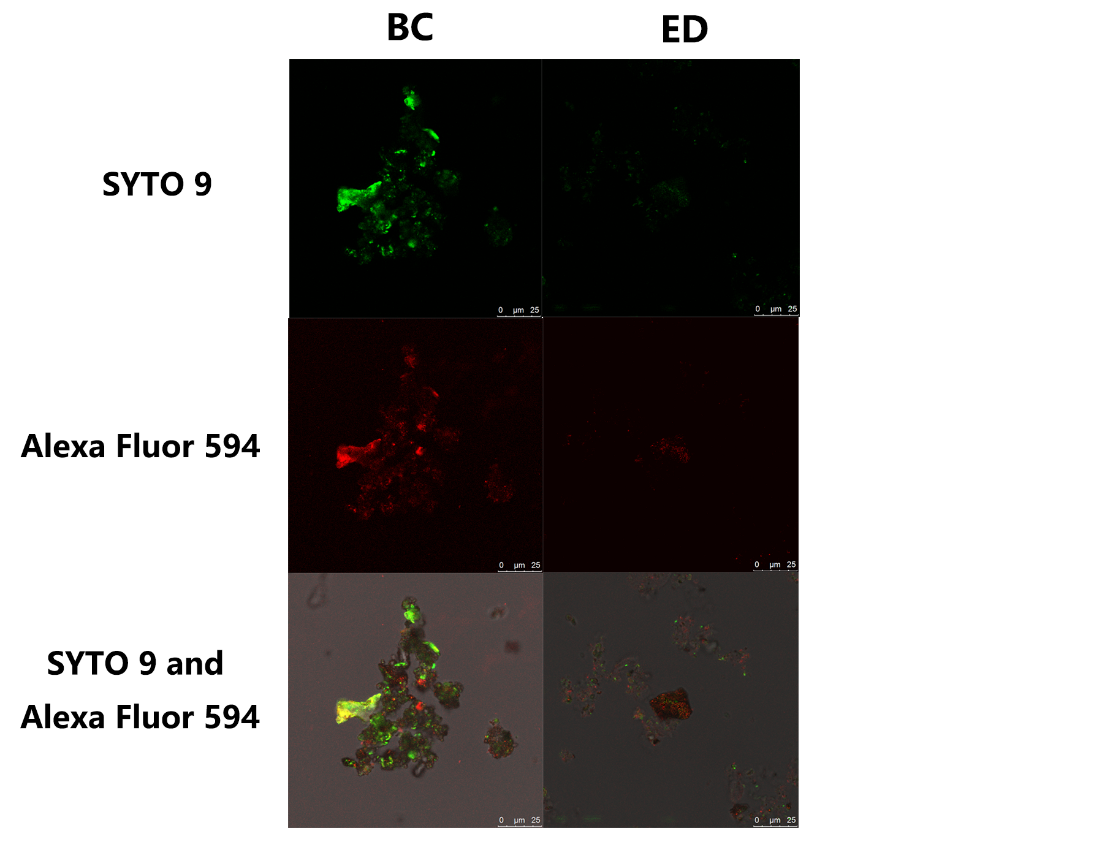


Figure CLSM analysis of attached cells and EPS on the mineral surface under different systems. BC blank control system; ED EPS deficient system. The leached ore samples were collected at different bioleaching periods. 20 μM SYTO 9 (L13152, Invitrogen, USA) was mixed with the sample (*λ_ex_*: 485 nm and *λ_em_*: 498 nm) with 200 μg/mL Alexa Fluor 594 ConA (C11253, Invitrogen, USA) (*λ_ex_*: 590 nm and *λ_em_*: 617 nm). After incubation in the dark for 30 min, the ore sample was collected by centrifugation, washed with 1 mL of PBS solution for 30 min. The treated ore sample was then incubated with PBS solution for 30 min and observed via CLSM (TSC SP8, Leica, Germany).
